# A dynamic approach to assess international competitiveness of Vietnam’s garment and textile industry

**DOI:** 10.1186/s40064-016-1912-3

**Published:** 2016-02-27

**Authors:** Huong Thanh Vu, Lam Cat Pham

**Affiliations:** Faculty of International Business and Economics, University of Economics and Business, Vietnam National University – Hanoi, 144 Xuan Thuy, Cau Giay, Hanoi, Vietnam

**Keywords:** Garment and textile, Vietnam, China, International competitiveness, Generalized Double Diamond Model

## Abstract

Garment and textile (G&T) industry has been playing as a driving force for the socio-economic development of Vietnam. With the international integration process and rising challenges from the global market, there is a need to examine international competitiveness of Vietnam’s G&T industry to find out what Vietnam should focus on to enhance its position in the global market place. This paper, by using the Generalized Double Diamond Model (GDDM), analyzed international competitiveness of Vietnam’s G&T industry and compared it with China. The results showed that Vietnam was less competitive than China in all four attributes of the GDDM. The lowest competitiveness of Vietnam in comparison with China was Related and Supporting industries, followed by Factor Conditions. Therefore, the paper argued that although Vietnam should improve all of the four attributes in the long term, Vietnam must put a high priority on developing Related and Supporting Industries and then enhance Factor Conditions while maintaining its strengths over China in terms of G&T export growths and favorable business context.

**Electronic supplementary material:**

The online version of this article (doi:10.1186/s40064-016-1912-3) contains supplementary material, which is available to authorized users.

## Background

In Vietnam, garment and textile industry (G&T) has been a key exporting industry and contributed considerably to the social and economic development. In 2014, G&T was the second biggest exporting industry and contributed 11.51 % to the total GDP of Vietnam (General Departnment of Vietnam Customs 2015). In the international market, with the share of nearly 3 % in the world G&T exports in 2013, Vietnam has become the 7th biggest G&T exporter after China, the EU, India, Turkey, Bangladesh and the US (ITC 2015). Together with the increasing integration in the global marketplace, Vietnam’s G&T industry is facing with fiercer competition from other competitors. In order for the G&T industry of Vietnam to compete successfully and move up in the international G&T market, the understanding of its international competitiveness is of great importance.

International competitiveness is viewed as a strategic phenomenon inherent in the fields of international marketing, international business and international management, and refers to the attributes that make organizations more competitive than others in the global market. Organizations can be understood broadly as a region, a nation, an industry or perhaps a strategic group. Therefore, the term international competitiveness is a multi-level phenomenon working in the global market (Hult 2012). Porter (1990) argued that the competitiveness is created, not inherited and claimed that the source of competitiveness is the competitive advantage, which is created and sustained through a highly localized process. Porter (1990) described the competitive advantage as four main attributes that allow an organization to outperform its competitors. Those attributes, individually and as a system, constitute the Diamond Model of national advantage, which serves as a playing field that each nation establishes and operates for its industries. The competitiveness will increase when each of these attributes is improved. Based on Porter (1990) and D’Cruz and Rugman (1993), the Generalized Double Diamond Model (GDDM) introduced by Moon et al. (1995, 1998) viewed international competitiveness as a broader term to incorporate multinational activities and government in the Diamond Model. At the sector level, D’Cruz (1992) argued that international competitiveness of an industry is the collective ability of firms in that sector to compete internationally. According to Momaya (1998), international competitiveness at industry level is often considered as the results of strategies and actions of firms operating in that sector. Competitiveness is also represented by the relative productivity and its ability to create value added. It allows an industry to maintain and improve position in the global market and can only be assessed by comparing with the same industry in another country (Depperu and Cerrato 2005). With all the ideas above, international competitiveness of an industry in short can be understood as its ability to compete internationally and can be measured through different attributes in comparison with the same industry in other nations.

Even though Vietnam’s G&T industry is a common topic for researchers in Vietnam, the previous literature on its international competitiveness is limited. The past studies examining international competitiveness of Vietnam’s G&T adopted three main approaches namely the value chain, strengths, weaknesses, opportunities and threats (SWOT) and the Diamond Model of Michael Porter. Vitas (2006) used SWOT and value chain analysis to assess Vietnam’s G&T international competitiveness through a great deal of indicators, for example cost, production time, customs procedures, policies and supporting industries in comparison with some main competitors such as China, India, the US, Bangladesh and Thailand. A mixed picture was pointed out in the paper and eventually it was not clear of whether Vietnam’s international competitiveness in G&T industry was higher or lower compared to that of other competitors. Also using the value chain approach, Truong et al. (2010), Dang and Dinh (2011), and Luong (2012) argued that Vietnam’s G&T international competitive was still low because of its participation in the lowest end of global G&T value chain. Nguyen (2012) using SWOT and the Diamond Model, and IPP and CIEM (2013) adopting the value chain and the Diamond Model drew out the same conclusions that Vietnam was at low international competitiveness mainly because of its dependence on the outside raw material, weak supporting industries and low productivity. Asian Foundation and CIEM (2012) also found out that Vietnam’s G&T international competitiveness was modest and affected by tariff, customs, financial policies, labor, technology, materials input, market, and products quality. The previous studies recommended that in order to improve the international competitiveness of Vietnam’s G&T industry, Vietnam should increase the localization rate, develop Vietnam’s brand name, and increase the added value to move up in the value chain.

The nature of international competitiveness of an industry as stated above is its ability to compete internationally and must be assessed by comparing with the same industry in other countries. One common point of all previous papers relating to international competitiveness of Vietnam’s G&T is that although they were informative to describe the current development of this industry, they failed to measure or quantify international competitiveness of Vietnam’s G&T aggregately in indexes and therefore it is hard to position Vietnam’s international competiveness in the global marketplace. In addition, the past studies proposed a wide range of measures for Vietnam’s G&T industry but the priority measures were unclear. To fill this gap, the paper concentrates on analyzing and measuring international competitiveness of Vietnam’s G&T industry by using the GDDM. With this methodology and framework, the contribution of this paper is twofold. Firstly, the paper develops a specific framework for assessing international competitiveness of G&T industry. Secondly, this is the pioneering study adopting the GDDM to examine international competitiveness of an industry in Vietnam.

In this paper, the GDDM, with the analysis of both domestic and international attributes, helps answer how internationally competitive Vietnam’s G&T is quantitatively compared to the benchmark country, China, and draw out better implications for Vietnam to improve international competitiveness of G&T industry. The paper is structured as below. After the introduction, the second part introduces the framework used while the third part explains the methodology and selection of proxies for the GDDM. In the next part, the paper presents the main results on international competitiveness of Vietnam’s G&T and the final part points out some conclusions and implications, which are essential for Vietnam’s G&T industry to enhance its international competitiveness in the future.

## Analytical framework

Among the models that are adopted to explain nation or industry competitiveness, the widely used one is Michael Porter’s Diamond Model introduced firstly in Porter (1990). According to this model, four main attributes that underlie conditions or platform for determination of the national competitive advantage are “Factor conditions”, “Demand conditions”, “Related and Supporting Industries”, and “Firm Strategy, Structure and Rivalry”. Porter (1990) also proposed government policies and chance as exogenous shocks, which supported the whole system of national competitiveness with four above-mentioned attributes.

In spite of being influential and widely used, Michael Porter’s Diamond Model of competitiveness still has some severe limitations. Firstly, the Diamond Model leaves out the multinational activities such as inbound and outbound foreign direct investment (FDI) and is mainly fixed on a large home base country (Cartwright 1993; Cho 1994; D’Cruz and Rugman 1993; Dunning 2003; Moon et al. 1998; Williams and Morgan 2010). Secondly, the Diamond Model is successful at explaining international competitiveness of big countries like the US and Japan, but not appropriate to analyze the competitiveness of advanced, smaller and open countries like Canada (D’Cruz and Rugman 1993). Therefore, Rugman and Verbeke (1993) proposed an alteration to Porter’s Diamond Model, which is called the Double Diamond Model (DDM). This model covers the same four groups of attributes of competitiveness as the Diamond Model but takes into account the activities of multinational enterprises, which have to rely on both home-base and foreign determinants to sustain its competitive advantage, and suggests that managers should build upon both domestic and foreign diamonds to become globally competitive in terms of survival, profitability and growth (Liu and Hsu 2009). However, there are still problems with the DDM. Although this model can be used to explain quite well the cases of countries like Canada and New Zealand, it fails to analyze the competitiveness of all other small open countries such as Korea and Singapore (Son and Kenji 2013). In fact, multinational firms from these small countries have to rely not only on domestic determinants, but also on the resources and markets internationally and especially are likely to link more with global than domestic industrial structure. Therefore, Moon et al. (1998) developed the GDDM, which is suitable for all small open economies (Balcarová 2010; Son and Kenji 2013).

**Fig. 1:**
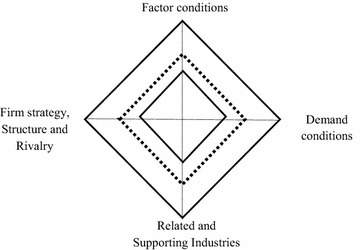
The Generalized Double Diamond Model. *Source*: Moon et al. (1998)

The GDDM consists of two main diamonds (Fig. 1). The inner represents the Domestic Diamond, which is similar to the diamond of Michael Porter. The outside one is the International Diamond, which represents all four attributes in international context. In these two diamonds, chance is included and treated as exogenous variables. Government influence, on the other hand, is included as an important endogenous variable that directly influences all four determinants. The dotted Global Diamond, between the Domestic and International Diamond, represents international competitiveness of an industry as determined by both domestic and international parameters. Difference between the Global Diamond and the Domestic Diamond of Michael Porter is the result of integrating multinational activities into the model.

Compared to the Diamond Model of Porter (1990), this model has three important extensions: (1) incorporates the multinational activities, (2) be able to function the competitiveness paradigm which allows a comparison of size and shape of the Domestic and International diamonds and (3) fits all small open nations in which firms are likely to be concerned more with global than domestic industrial structure (Sardy and Fetscherin 2009; Son and Kenji 2013). With these extensions, this model has been proven to be more generalized and useful in analyzing international competitiveness of different countries at multi-levels, nationally or industrially. For example, Sardy and Fetscherin (2009) analyzed and compared international competitiveness of automotive industry between China, India and South Korea, three countries at different sizes but to be three of ten biggest automotive producers in the global market place. Results from the Global Diamond of these countries pointed out that China’s automotive industry was as competitive as South Korea’s in terms of Factor Conditions, Demand Conditions and Related and Supporting Industries but was more competitive than India’s. Son and Kenji (2013) adopted the GDDM to compare international competitiveness of Korean and Japanese fashion industries based on 32 proxies for four attributes including Factor conditions, Demand conditions, Related and Supporting Industries and Firm Strategy, Structure and Rivalry. They collected secondary data from different sources of Korea and Japan in different years from 2007 to 2010, then calculated international competitiveness indexes of two nations and found out that Korea was less competitive than Japan. While Son and Kenji (2013) applied the GDDM for industrial competiveness comparison, Liu and Hsu (2009) used this model to analyze the overall competitiveness of two open economies including Taiwan and Korea. The results showed that Taiwan was superior to Korea in all attributes except for Demand Condition in the Domestic Diamond. With the same objectives of comparing China and Korea’s international competitiveness of fashion industries, Kim et al. (2006) and Son et al. (2007) adopted the GDDM and suggested an entry strategy for the Chinese fashion market. From the previous typical literature, it can be seen that the GDDM can provide a better insight of international competitiveness due to incorporating multinational activities into the Diamond Mode and might be used to compare competiveness of at multi-levels.

## Methodology and data

The objective of this paper is to assess international competitiveness of Vietnam’s G&T industry. In this regard, a methodology that helps quantifying Vietnam’s competitiveness and allowing a comparison of this competitiveness level to that of a benchmark country will be required. The GDDM is therefore selected as this model satisfies these two requirements. The model is also proved to be suitable for an open and developing country like Vietnam.

The comparison country chosen in this study was China, which is not only Vietnam’s neighboring country sharing the similar cultures and traditions, but also the top G&T exporter in the world. Domestically, the Vietnamese G&T industry competes with China’s in providing G&T products to Vietnam’s citizens. Internationally, the position that China’s G&T industry holds at the moment is the one that Vietnam’s G&T industry heads for. Moreover, both China and Vietnam are among top G&T exporters in the international marketplace. Comparing the competitiveness of Vietnam’s G&T industry to that of China is necessary for Vietnam to know where it is at the moment in the race with China. Therefore, although China is the second biggest country in the world in terms of GDP, the comparison of two nations at G&T industry level is acceptable as long as cautious analysis is taken when examining proxies that are not at industry level like total population and total GDP.

The key issue is the choice of proxies capturing four attributes that are assessed in the GDDM. The GDDM is developed from the original Diamond Model of Porter, which consists of four groups of attributes namely Factor conditions, Demand conditions, Related and Supporting industries, and Firm Strategy, Structure and Rivalry. In fact, the model has generated over 100 proxies that are used to capture international competitiveness. However, like other previous studies by Rugman and Verbeke (1993), Sardy and Fetscherin (2009), Balcarová (2010), Williams and Morgan (2010), and Son and Kenji (2013), this paper does not cover all proxies but chooses certain proxies which best capture international competitiveness of studied industry. Totally, 27 proxies that describe four attributes, taking into consideration G&T industry-related features, were selected to act as determinants of the model (Table 1).

**Table 1:** Variables and proxies of GDDM for Vietnam’s G&T industry in comparison with China’s

Attributes	Variables	Proxies
Factor Conditions	Domestic	
	Basic factors	Wage of worker in G&T industry (USD/h)
		Number of workers and laborers in G&T industry (million people)
		Labor productivity in G&T industry (shirts/worker/day)
	Advanced factors	R&D expenditure (% of GDP)
	International	
	Advanced factors	Manufacturing inward FDI flows (billion USD)
		Manufacturing outward FDI flows (billion USD)
Demand Conditions	Domestic	
	Size	Total population (million people)
		GDP (billion USD)
		Employment rate (%)
	Sophistication	GDP per capita (USD)
		Household rate of expenditure on G&T out of gross income (%)
		Educational index
	International	
	Size	Total export value of G&T industry (billion USD)
		Average export growth rate of G&T industry (%)
Related and Supporting Industries	Domestic	
	Supporting Industries	Cotton output (1000 t)
		Yarn output (million tons)
	Supporting infrastructures	Rail lines (total route—km)
		Roads, paved (% of total roads)
		ICT index
	International	
	Supporting industries	Cotton exports (1000 t)
		Yarn and fabric exports (billion USD)
	Supporting infrastructures	Container port traffics (TEU: 20 foot equivalent unit)
		Air transport (registered carrier departures worldwide)
Firm Strategy, Structure and Rivalry	Domestic	
	Rivalry	Intensity of local competition
	Business context	World Bank DTF points
	International	
	Rivalry	Market share of the country in G&T global market (%)
	Business context	Average import tariff rate faced by G&T industry (%)

Sources of data are provided in Additional file 1

*Source:* Developed by the authors based on Rugman and Verbeke (1993), Sardy and Fetscherin (2009), Balcarová (2010), Williams and Morgan (2010), Son and Kenji (2013)

### Factor Conditions

According to Porter (1990), the domestic Factor Conditions include both basic and advanced factors. Basic factors refer to natural resources, climate conditions, location, unskilled labor, and semiskilled labor that are inherited and require little investment to be utilized in the production process. Advanced factors such as highly skilled workers, highly educated personnel, and Research & Development (R&D), on the other hand, are created and upgraded through reinvestment and innovation.

Four proxies were used to assess the domestic Factor Conditions (Table 1). Because G&T is a labor-intensive industry, the paper puts priority to select labor-related proxies including (1) the wages of G&T workers, (2) the number of workers and laborers in G&T industry and (3) the labor productivity in G&T industry to represent the basic factor conditions. Low wages and high number of workers represent cheap labor and labor abundance, implying a possible motive for expansion of the industry and therefore high competitiveness (Brown and Sessions 2001; Pizer 2000; Sardy and Fetscherin 2009). Some studies also showed that high productivity captures high competitiveness of the industry (Daniel 2000; Han et al. 2015). As variables for advanced factor conditions, (4) R&D expenditures were selected as a proxy for future growth and innovation of the industry.

International factors were assessed based on two aspects: (1) inward and (2) outward FDI (Table 1). While the inward FDI shows the foreign investment in domestic market, the outward FDI represents the outward investment made by domestic firms. Because the data of inward and outward FDI by specific sector were not available in Vietnam, the data of manufacturing inward and outward FDI were used instead. The more inward and outward manufacturing FDI are, the higher competitiveness the manufacturing including G&T is in the international market (Moon and Youn 2010).

### Demand Conditions

Porter (1990) emphasized the role of size and sophistication of Domestic Demand in shaping the competitiveness. While size of home demand forces firms to expand their production to take advantage of economics of scale, sophistication of demand drives firms to continuously change and innovate to meet the high demands in terms of product quality and varieties (Smit 2010).

In this paper, (1) the total population, (2) GDP and (3) the employment rate were used to represent the size of domestic market demand (Table 1). As G&T is a necessity in daily life, the population and GDP are good proxies to represent the domestic demand for G&T products. Meanwhile the employment rate is an additional index to represent the extent to which people can afford satisfy their demand. According to Son and Kenji (2013), the sophistication of clothes buyers is related to fashion buying behaviors such as expenditure on clothes and the frequency of purchasing clothes. Hirschman (1980) and Barnes and McTavish (1983) also argued that consumer sophistication is driven by the fact that the consumers are better informed and educated. Therefore, (4) GDP per capita, (5) household rate of expenditure on G&T products and (6) educational index were used as proxies for sophistication of demand.

The international demand factors were determined through size of the international market, which can be represented by (1) total G&T export value and (2) growth rate of G&T export value (Table 1). The higher export values and growth rates shows the higher and more stable demand for a country’s product, implying higher competitiveness. A country normally exports to multiple foreign countries; therefore, there is no appropriate proxy for assessing the sophistication of international demand (Sardy and Fetscherin 2009).

### Related and Supporting Industries

Related and Supporting Industries are essential for ability of an industry to compete in the international market as a industry is more likely to be successful if its supporting industries have a competitive advantage (Sardy and Fetscherin 2009). According to Porter (1990), Related and Supporting Industries attribute refers to the presence or absence in the nation of related industries and suppliers that are internationally competitive. They include the upstream and downstream firms as well as the supporting infrastructure like transportations and communication involved in the value chain. The upstream industries include firms that produce input materials and the downstream ones that are in charge of distributing G&T products to final customers. For Domestic Diamond, this paper used (1) the amount of cotton produced and (2) the amount of yarn produced as proxies to reflect the domestic downstream industries because cotton and yarn are two important inputs of G&T industry. In today’s globalization, transportation and communication are essential to promote the competitiveness of an industry (Hult 2012; Son and Kenji 2013; Williams and Morgan 2010). Accordingly, (3) rail lines and (4) the share of paved roads were used as indices for transportation while (5) ICT development index for communication (Table 1).

The international factors were analyzed based on the presence of internationally competitive Related and Supporting Industries. (1) Cotton exports and (2) yarn exports were used to indicate how strong these industries were in the global market through its expansion into related and supporting international industries (Table 1). These two proxies were adopted also because cotton and yard consumption is heavily driving G&T industry. In addition, infrastructure for international transportation are important for improving international competiveness because they facilitate international trade transactions and increase the levels of multinational activities with higher efficiency (Daniel 2000; ITS Global 2008). Therefore, (3) container port traffics and (4) the number of registered air departures worldwide were used to represent the ability to ship goods abroad. While container port traffic measures the flow of containers from land to sea transport modes in 20-foot equivalent units (TEU), the registered air departures provides information on the number of domestic takeoffs and takeoffs abroad of air carriers registered in the country.

### Firm strategy, structure and rivalry

This attribute reflects the context in which firms are created, organized and managed and the domestic competition environment (Porter 1990). While Porter (1990) focused on the rivalry and considered it as the most critical driver of competitive advantage of a country or an industry, national competitive advantages can be also gained from a good business context (Liu and Hsu 2009).

Competition is the spur that drives firms to look for ways to save costs, increase efficiency and encourage innovation, which are all means of increasing competitive advantages (ITS Global 2008; Mitschke 2008). In this paper, (1) intensity of local competition index^1^ was used to show the level of domestic competition while the domestic business environment was represented by (3) DTF (Distance to Frontier). DTF is a measurement developed by the World Bank in Doing Business Report to show the distance of each economy to the “frontier,” which represents the best performance observed on each of the indicators^2^ across all economies. Value 0 shows the lowest performance and 100 reveals the highest performance. Therefore, DTF is a good proxy for domestic business context of one country in comparison with others.

The international factors were also analyzed in two aspects namely rivalry and business context. (1) Market share of the country in G&T global market was used for rivalry proxy and (2) the average import tariff rate representing international business context (Table 1). The higher market share but lower average tariff rate implies higher competitiveness.

To measure 27 selected above-mentioned proxies, data used in this paper were the secondary data derived from various sources of Vietnam, China, and the international organizations such as the World Bank, United Nation Development Program (UNDP), International Trade Center, International Telecommunication Union and the United States Department of Agriculture. The latest available data for both Vietnam and China were used for each proxy. One point worth commenting is that the paper aims at assessing international competitiveness of Vietnam’s G&T industry through comparing it with China’s. Therefore, data of all proxies are not necessary to be collected for 1 year but data for a proxy must be in 1 year for both nations. This is also data selection approach used in previous literature by Moon et al. (1998), Liu and Hsu (2009), Sardy and Fetscherin (2009), Balcarová (2010), Williams and Morgan (2010), Son and Kenji (2013).

After data for the above-mentioned proxies were collected, they were then translated into scores to quantify international competitiveness of G&T industry in Vietnam and the benchmark nation, China. The method of score translation used in this study was similar to the one used in Rugman and Verbeke (1993) and Sardy and Fetscherin (2009). Firstly, the value of 100 was set for a benchmark country in all proxies, and based on that, the value of each proxy in another country would be calculated accordingly. In this paper, the value of 100 was set for all proxies of China, the country of comparison and the competitiveness index of Vietnam’s G&T industry was then calculated accordingly. If a proxy where the higher value indicated more competitiveness, data of China were taken as the base or denominator in calculations. For instance, in the case of labor productivity in G&T industry, a Vietnam’s G&T worker can produce seven shirts/day while that number of Chinese worker is 16 (Table 2). Because the higher productivity indicates more competitiveness, the competitiveness value of Vietnam for this proxy is 43.75 % (7/16) (Additional file 2). In contrast, if a proxy where the higher value indicated less competitiveness, data of China were be taken as the numerator. For instance, wage of a worker in G&T industry of Vietnam is 0.74 compared to 2.65 of China (Table 2). Then, the competitiveness index of Vietnam for this proxy is 358.11 % (2.65/0.74) (Additional file 2). Secondly, for each country, the index values of Domestic Diamond and International Diamond were calculated by using the simple average of all selected proxies and variables because they were considered equally important in determining international competitiveness. Thirdly, the global indexes were calculated by taking the simple average of the Domestic and International indexes, representing international competitiveness of Vietnam G&T industry.

**Table 2:** Descriptive data for GDDM of Vietnam and China’s G&T industry

Attributes	Variables	Proxies	Vietnam	China
Factor Conditions	Domestic		0.74	2.65
	Basic factors	Wage of G&T worker (USD/h) (2014)		
		Number of workers and laborers in G&T industry (million people) (2013)	2.5	23
		Labor productivity in G&T industry (shirts/worker/day) (2012)	7	16
	Advanced factors	R&D expenditures (% of GDP) (2013)	1.48	2.08
	International			
	Advanced factors	Manufacturing inward FDI flow (billion USD) (2013)	17.14	455.54
		Manufacturing outward FDI flow (billion USD) (2013)	1.96	71.97
Demand Conditions	Domestic			
	Size	Total population (million people) (2014)	90.7	1364.3
		GDP (billion USD) (2014)	186.2	10,360.1
		Employment rate (%) (2013)	76.0	68.0
	Sophistication	GDP per capita (USD) (2014)	2052.3	7593.9
		Household rate of expenditure on G&T out of gross income (%) (2010)	3.19	6.96
		Educational index (2013)	0.51	0.61
	International			
	Size	G&T Export value (billion USD) (2014)	26.18	287.59
		Average G&T export growth rate (%) (2012–2014)	16.17	6.20
Related and Supporting Industries	Domestic			
	Supporting Industries	Cotton output (1000 t) (2014–2015)	1.36	6532
		Yarn output (million ton) (2013)	0.72	32
	Supporting infrastructures	Rail lines (total route—km) (2012)	2347	66,298
		Roads, paved (% of total roads) (2010)	47.6	60.9
		ICT index (2013)	4.09	4.64
	International			
	Supporting industries	Cotton exports (1000 t) (2014)	0.00	16,000
		Yarn and Fabric exports (billion USD) (2013)	3.26	106.90
	Supporting infrastructures	Container port traffics (TEU: 20 foot equivalent unit) (2013)	8,121,019	170,080,330
		Air transport (registered carrier departures worldwide) (2014)	144,630	3,356,756
Firm Strategy, Structure and Rivalry				
	Domestic			
	Rivalry	Intensity of local competition (2013–2014 weighted average)	5.10	5.40
	Business context	World Bank DTF point (2014)	64.42	62.58
	International			
	Rivalry	Market share of the country in G&T global market (%) (2014)	3.16	34.69
	Business context	Average import tariff rate faced by G&T industry (%) (2013)	12.4	12.8

*Source*: Synthesized and calculated by authors

Calculation of competitiveness index of each proxy was shown in Addition files 2, 3, 4, and 5

Calculation of competitiveness index of the GDDM model was shown in Additional file 6

## Results and discussion

From descriptive data for Vietnam and China’s G&T industries shown in Table 2, the paper calculated and analyzed competitiveness indexes of two countries. Results of the Domestic Diamond, International Diamond and Global Diamond are presented bellowed.

### Domestic Diamond

**Fig. 2:**
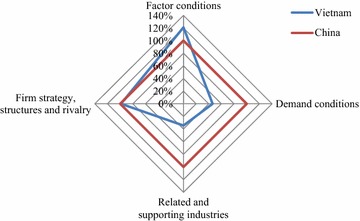
Domestic Diamond of Vietnam’s G&T industry in comparison with China’s

Except for Factor Conditions, Vietnam’s G&T industry was less competitive than China’s in all domestic determinants (Fig. 2). The reason behind the higher measurement of Vietnam’s factor conditions was the abundant source of unskilled labor with low costs. Vietnam in 2014 was the 14th most populous country in the world and had a golden population structure with the number of working people being twice as much as the number of dependent people (CIA 2014). However, more than 80 % of labors in Vietnam were untrained (General Statistics Office 2015) and therefore were low paid. According to Werner International, the average wages for labors in Vietnam’s G&T industry in 2014 was only 0.74 USD/h, the lowest among Southeast Asian nations and 3.5 times lower than that in China (Table 2). In addition, the fee for labor training was also low in Vietnam due to the fact that most of unskilled labors in Vietnam’s G&T industry learnt how to work by doing themselves and receiving advices from more experienced workers. G&T is a labor-intensive industry and thus the labor cost has a substantial share in the total cost (IPP and CIEM 2013), leading to big cost competitiveness for Vietnam’s G&T industry. This cost competitiveness to some extent compensated for Vietnam’s weaknesses in productivity (Yen 2012), the number of workers in G&T industry (Bui 2014, Kane 2014) and the amount of R&D investment compared to China. As the result, Vietnam surpassed China in Factor Conditions.

The domestic Firm Strategy, Structures and Rivalry of Vietnam were only about 1 % less competitive to China’s (Fig. 2). Thanks to improvement in business environment, Vietnam’s business context was 102.94 % better than China and the level of local competition in Vietnam was relatively high, equal to 94.44 % of China’s (Schawab and Martín 2014) (Additional file 5), implying a fierce competition in Vietnam’s domestic market. The first source of this competition came from the international integration policy of Vietnam, in which Vietnam opened market to foreign G&T producers to make investment in Vietnam. The second source was low barrier to enter but significant barrier to exit G&T industry. While the low technological requirements and abundant labor made it easier for newcomers to enter G&T industry in Vietnam, the specialized machines in contrast put the existing firms in a difficult situation to get out of the industry. The other source of competition came from the foreign rivalry in selling G&T products. The foreign G&T firms in Vietnam with higher technology, larger scale and bigger experience often achieved the higher share of G&T exports (Bui 2014). The low diversity of Vietnam’s G&T products resulting from the insufficient R&D investment also made the competition among domestic firms more drastic. Totally, China was more competitive than Vietnam in this attribute but the gap between two countries was modest.

The domestic Demand Conditions of Vietnam were far less completive than that of China (Fig. 2) because it reported lower measurements relating to five of six proxies, namely total population, GDP, GDP per capita, rate of expenditure on G&T and educational index (Table 2; Additional file 3). However, even though Domestic Conditions of Vietnam was less competitive, there were signs of possibility for Vietnam to improve its competitiveness in the future. The domestic market in Vietnam was small compared to China with the population of being equal to 6.65 % of China’s, but Vietnam was a promising market for the development of G&T industry. In fact, Vietnam was a diverse market with increasing demand for G&T product. While the diversity was shown by 54 ethnic groups with different clothing cultures and preferences, the increasing demand was indicated by the high growth rate of domestic demand. According to the survey of household living standards conducted by the General Statistics Office of Vietnam in 2012, the annual average expenditures on garments, hat, shoes and sandals of a Vietnamese household increased more than 1.5 times from 2008 to 2012. This resulted from the increase in the employment rate and GDP per capita in Vietnam during this period. Beside the size, the sophistication of domestic demand also showed some positive signs. Despite being weak compared to China, the education in Vietnam progressed in the recent years, resulting in an increase in Education Index of Vietnam from 0.49 in 2008 to 0.61 in 2013.

China was superior to Vietnam in all five proxies of Related and Supporting Industries. Vietnam’s competitive value of this attribute was only equal to 34.42 % of China (Fig. 2). Though the supporting infrastructures such as transportation and communication have recently experienced improvements in Vietnam, problems are still being found in the upstream and downstream industries.

Regarding the upstream industries, both Vietnam’s cotton and yarn industries are revealing big weaknesses. Vietnam has consistently confronted the lack of cotton and yarn for G&T production. In Vietnam, only 2.5 thousand hectares was used for cotton cultivation in marketing year (MY) 2014/2015 compared to 4.4 million hectares in China because of unfavorable weather condition and restricted agricultural land. Therefore, cotton output of Vietnam reached 1.36 thousand tons, equivalent to only around 0.02 % of that of China (Additional file 4). The situation is forecasted to be worse because of the decreasing cotton planted area in the years to come and the low productivity of cotton farmers, resulting in the fact that Vietnam’s G&T industry will rely more heavily on cotton imports. The domestic cotton production met only around 2 % of total domestic demand and the rest of 98 % must be imported (Vu 2014). Regarding the yarn industry, there has been a paradox. Though the yarn industry experienced some developments when the total output in 2013 rose to 720 thousand ton, equal to 2.1 % of the world total yarn output, only 30 % of the output could be used domestically while the remaining part had to be exported (Vo and Wilder 2015). The reason behind this paradox was the low quality and the lack of diversity in Vietnam’s yarn. Vietnam’s yarn industry has just focused on low-end products, which cannot satisfy the demand of domestic G&T industry. In comparison with China, yarn output of Vietnam was only equivalent to 2.25 % of that of China. China has always been the world’s biggest producer of cotton and yarn due to special status of its agriculture and superiority in resources for G&T industry (Yuan and Xu 2007; Meador and Wu 2014).

While Vietnam’s upstream industries were really weak compared to China’s, the downstream industries that are related to marketing and distribution have also experienced the same situation. 73 % of Vietnam’s G&T exports applied cut–make–trim (CMT) method, by which the Vietnamese G&T firms received orders from their partners abroad, manufactured and then sent back the final products, which would be distributed and sold by foreign partners. Or Vietnam’s G&T firms exported products to destinations as instructed by foreign partners. Therefore, marketing and distribution network of Vietnam’s G&T enterprises has been underdeveloped and relied largely on foreign distributors (Bui 2014; Vu 2014). Vietnam’s G&T enterprises have also participated in the lowest end of global G&T value chain (Truong et al. 2010; Dang and Dinh 2011; Luong 2012). China in contrast gradually moved up in G&T global value chain by shifting to the higher value-added stages rather than CMT. The Chinese G&T companies conducted vertical integration and were able to offer everything from design input to packaging, customs and shipping services. The Chinese companies were also innovative in choosing their business model for their own markets (McNamara 2008).

The weak upstream and downstream industries of Vietnam together with the loose connectivity between them have also resulted in the lack of G&T clusters in Vietnam. In fact, Vietnam had only a handful number of G&T clusters with the biggest one located in the south. This cluster was the result of the co-operation between Ho Chi Minh City, Dong Nai province and Binh Duong province, contributing 56.4 % to total G&T output, 39.4 % to total export value, and 30 % to total labor in G&T industry in 2011 (IPP and CIEM 2013). Vietnam was therefore far behind China with 151 G&T clusters by May 2011 (EUSME Centre 2011). As forming a cluster is vital in improving international competitiveness of an industry (Porter 1990), the lack of G&T clusters has deteriorated international competitiveness of Vietnam’s G&T industry considerably in comparison with China. With the above-mentioned reasons, Related and Supporting Industries become the most important and different attribute affecting the competitiveness of Vietnam’s G&T in Domestic Diamond.

### International Diamond

**Fig. 3:**
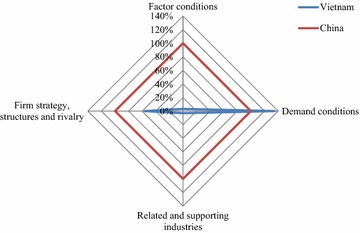
International Diamond of Vietnam’s G&T industry in comparison with China’s

The International Diamond of Vietnam in G&T industry was much worse than China’s. China surpassed Vietnam in all attributes, except for Demand Conditions (Fig. 3).

Vietnam showed better Demand Conditions than China in the international context (Fig. 3) because G&T exports of Vietnam witnessed a high growth rate of more than 16 % compared to only 6.2 % of China in the period 2012–2014 (Table 2). There were two reasons for this miraculous rate. Firstly, the world economy recovered from the global crisis, leading to higher income and demand for goods and services all over the world. Secondly, Vietnam took well advantage of the EU–Vietnam Partnership and Cooperation Agreement (PCA) signed in 2012 and the EU’s debt crisis, when the EU’s demand for luxury goods decreased but for necessity goods like food and clothes increased. In addition, in this difficult time, the EU’s consumers had a tendency to come back to products with reasonable prices and quality like those made by Vietnam (Vietnam Trade Promotion Agency 2013). In 2012–2014, when the Eurozone was deep in the debt crisis, the growth rate of Vietnam’s G&T exports to the EU reached more than 8.4 % while this rate for China was −2.3 %. Besides, Vietnam-Japan Economic Partnership Agreement, the newly signed Vietnam-Korean FTA and the forthcoming European Union and Vietnam FTA (EVFTA) are also motives for Vietnam to expand exporting G&T products to these key partners. Therefore, though the absolute exports of Vietnam’s G&T industry were still low compared to China’s, the remarkable growth rates made Vietnam more competitive than China in international Demand Conditions attribute.

Ranking second for Vietnam in the International Diamond was Firm Strategy, Structures and Rivalry attribute (Fig. 3). Vietnam reported lower measurement in international rivalry but exceeded China in business context (Table 2; Additional file 5). With the efforts of Vietnam’s government to integrate into the world economy through accessing the World Trade Organization, and signing ten multilateral and bilateral FTAs up to now (Vu and Nguyen 2015), the tariff faced by Vietnam’s G&T producers in the global market considerably reduced and was lower than that faced by China. Until now, the average tariff faced by Vietnam in the field of G&T was 12.4 % compared to 12.8 % of China (Table 2). In the near future, when Trans-Pacific Partnership Agreement (TPP) and EVFTA, in which China is so far the outsider, are concluded, the tariffs faced by Vietnam’s G&T are likely to reduce substantially because TPP and EVFTA involve the most important G&T partners of Vietnam such as the US, the EU and Japan.

China overwhelmed Vietnam in the international Factor Conditions (Fig. 3) because of its high inward and outward FDI (Zhou and Leung 2015). The manufacturing FDI inflows in Vietnam equaled only 3.76 % and similarly the outward FDI of Vietnam was only 2.72 % as much as China’s (Additional file 2).

Similarly, the Related and Supporting Industries of Vietnam reported much lower measurements than China’s with all four proxies of being less competitive. Vietnam did not export cotton, and the yarn and fabric exports equaled only about 3 % of China’s (Additional file 4). The ability to transport goods to the international market was also weak because the index of container port traffic was equivalent to 4.77 % and the index of the air transport being 4.31 % of China’s. This attribute of Vietnam was the weakest in the GDDM model and the most difference between Vietnam and China, and therefore needs comprehensive attention to be improved in the future.

### Global Diamond

**Fig. 4:**
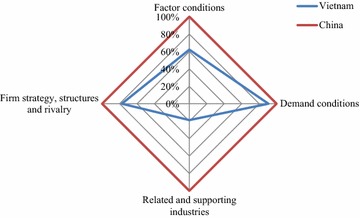
Global Diamond of Vietnam’s G&T industry in comparison with China’s

Integrating the Domestic and International Diamond provides the Global Diamond, which shows international competitiveness of Vietnam in G&T industry (Fig. 4).

International competitiveness of Vietnam in G&T industry was lower than China’s in all four GDDM attributes. The biggest gap and also the most important difference between Vietnam and China’s G&T competiveness can be seen in Related and Supporting industries, in which Vietnam was around 81 % lower than China (Additional file 6). In contrast, the lowest gap of 9.4 % was reported in terms of Demand Conditions. Factor Conditions and Firm Strategy, Structures and Rivalry of Vietnam were 37.9 and 22.6 % lower respectively. Therefore, in order for Vietnam to improve competitiveness in the global market, the Vietnamese government should put priority on long-term projects and policies to boost the Related and Supporting Industries, and pay more attention to enhance Factor Conditions for G&T industry.

## Conclusions

By using the GDDM, this paper analyzed international competitiveness of Vietnam’s G&T industry through comparing with China’s. The results showed that out of 27 proxies selected, Vietnam was superior to China in terms of only five proxies namely wages, employment rate, average G&T export growth, DTF point and average import tariff rate. As a result, the Domestic Diamond of Vietnam’s G&T industry showed lower measurements than China in all attributes, except for Factor Conditions. With the International Diamond, Vietnam was only more competitive in Demand Conditions. Totally, the Global Diamond showed that Vietnam’s G&T industry was far less internationally competitive than China’s in all four attributes.

The biggest gap between Vietnam and China’s international competitiveness in G&T industry was realized in Related and Supporting industries, in which Vietnam’s competitive index was more than 81 % lower than China’s. The low competitiveness of Vietnam originated from consistent problems of inadequate downstream and upstream industries including the insufficiency of cotton and yarn production; foreign-reliant G&T marketing and distribution; and weak transportation system for traded goods. Besides, the shortage of G&T cluster in Vietnam deteriorated the competitiveness of this industry internationally.

The second biggest gap was reported in Factor Conditions, in which Vietnam’s lower competitiveness came from the low labor productivity, limited R&D expenditure and especially far lowers inward and outward FDI in the manufacturing sector of Vietnam. Vietnam’s G&T industry development has so far relied heavily on low-cost labor that was not sustainable for future growth.

The third biggest gap between Vietnam and China in Global Diamond was Firm Strategy, Structures and Rivalry. Vietnam was better than China in business context with higher DTP point and lower average import tariff as a result of the dynamic international integration but China exceeded Vietnam in terms of intensity of local competition and global market share. Totally, Vietnam’s international competitiveness index was 38 % lower than that of China.

In the final attribute, Demand Conditions, Vietnam’s international competiveness index was approximate to China’s and only 9.4 % lower. This was due to Vietnam achieved a high and stable growth rate of G&T exports in the recent years and relatively high educational index. However, other criteria such as GDP per capita and rate of expenditure on G&T in Vietnam were lower than in China.

With all the above results, in order to improve international competitiveness of G&T industry, it is of great importance for Vietnam to enhance all of the four attributes. However, this paper argues that firstly Vietnam should put a high priority on promoting the Related and Supporting Industries. Develop cotton production, diversify and improve quality of yarn, support firms to build up its own distribution channel, set up G&T clusters and upgrade transportation for shipping goods abroad are urgent measures to enhance G&T Related and Supporting industries in Vietnam. Next is the task to improve Factor Condition by investing more in training G&T workers to enhance productivity and educating high skilled labors to improve R&D activities in the industry. Finally, Vietnam should put effort to maintain its current strengths over China in terms of G&T export growths and favorable business context through taking advantages of its existing FTAs and the promising EVFTA and TPP.

This paper has contributed to the existing literature by using the GDDM approach to analyze international competitiveness of Vietnam’s G&T industry. However, it still has limitation and can be improved in the future. In fact, due to limited data as well as differences in statistical system and methods between China and Vietnam, some data were not available for both nations. Therefore, some proxies for the whole manufacturing sector were used instead of those specifically for G&T industry including inward FDI, outward FDI and R&D expenditure. This shortcoming will be resolved in the future research when the data problem is addressed. Given Vietnam and China’s statistical system and methods are in status-quo, data limitation can be partly solved in future research through conducting survey or in-deep interview of Vietnam’s G&T enterprises and Vietnam Textile and Apparel Association to get G&T data on productivity, R&D expenditure and FDI.

Moreover, the future research can take into consideration some more G&T-related indicators such as design power in clothing and textiles (Factor Conditions); demanding needs of consumers, and preference for local brands in oversea market (Demand Conditions); G&T cluster, and educational facility related to G&T industry (Related and Supporting Industries); and the rate of added value in G&T industry (Firm Strategy, Structure and Rivalry) to provide better results on Vietnam’s competitiveness of G&T industry.

Finally, this paper chooses China, which is a developing but much bigger than Vietnam in terms of GDP, population and area, as the benchmark country to compare with Vietnam. In order to provide multi-dimensional analysis of Vietnam’s G&T international competitiveness, identify more precisely Vietnam’s G&T international competitiveness gap with its main competitors and more importantly recommend the progress measures for Vietnam to improve its international competitiveness, the future research can take into consideration of some other comparing nations with more similar size such as Bangladesh, Turkey and Cambodia.

## Additional files

10.1186/s40064-016-1912-3 Sources of data for the GDDM.
10.1186/s40064-016-1912-3 Competitiveness index of Factor Conditions.
10.1186/s40064-016-1912-3 Competitiveness index of Demand Conditions.
10.1186/s40064-016-1912-3 Competitiveness index of Related and Supporting Industries.
10.1186/s40064-016-1912-3 Competitiveness index of Firm, Strategy, Structure and Rivalry.
10.1186/s40064-016-1912-3 Competitiveness index of the GDDM.

## Abbreviations

DDM: the Double Diamond Model
EVFTA: the European Union and Vietnam Free Trade Agreement
FTA: Free Trade Agreement
G&T: garment and textile
GDDM: the Generalized Double Diamond Model
R&D: research and development
SWOT: strengths, weaknesses, opportunities and threats

## Footnotes

1 Intensity of local competition in an index used in the Global Competitiveness Report of World Economic Forum to measure the level of competition in the domestic market. 1 = not intense at all; 7: extremely intense.

2 Ten indicators are reported in Dong Business Report, namely starting a business, dealing with construction permits, getting electricity, registering property, getting credit, protecting minority investors, paying taxes, trading across borders, enforcing contracts and resolving insolvency.
