# Supplementary material for: A dynamic approach to assess international competitiveness of Vietnam’s garment and textile industry
Source: Springerplus. 2016 Feb 27;5:203. doi: 10.1186/s40064-016-1912-3 (PMC4769702; doi:10.1186/s40064-016-1912-3)
Supplement: Supplementary file 1 — 10.1186/s40064-016-1912-3 Sources of data for the GDDM. [file 40064_2016_1912_MOESM1_ESM.docx]

## Additional file 1 Sources of data for the GDDM

| **Attributes** | **Variables** | | **Proxies** | **Sources** |
| --- | --- | --- | --- | --- |
| **Factor Conditions** | *Domestic* | Basic factors | Wage of worker in G&T industry (USD/h) | Werner International <http://www.wernerinternational.com/index.html> |
|  |  |  | Number of workers and laborers in G&T industry (million people) | Vietnam GSO <http://gso.gov.vn/>  Bui (2014), Kane (2014) |
|  |  |  | Labor productivity in G&T industry (shirts/worker/day) | Yen Tuyet (2012) |
|  |  | Advanced factors | R&D expenditure (% of GDP) | Vietnam GSO <http://gso.gov.vn/>  National Bureau of Statistics of China <http://www.stats.gov.cn> |
|  | *International* | Advanced factors | Manufacturing inward FDI flows (billion USD) | Vietnam GSO <http://gso.gov.vn/>  Foreign Investment Agency of Vietnam  <http://fia.mpi.gov.vn>  National Bureau of Statistics of China <http://www.stats.gov.cn>  Zhou and Leung (2015) |
|  |  |  | Manufacturing outward FDI flows (billion USD) |  |
| **Demand Conditions** | *Domestic* | Size | Total population (million people) | World Bank <http://data.worldbank.org> |
|  |  |  | GDP (billion USD) | World Bank <http://data.worldbank.org> |
|  |  |  | Employment rate (%) | World Bank <http://data.worldbank.org> |
|  |  | Sophistication | GDP per capita (USD) | World Bank <http://data.worldbank.org> |
|  |  |  | Household rate of expenditure on G&T out of gross income (%) | World Bank Global Consumption Database <http://datatopics.worldbank.org/consumption/> |
|  |  |  | Educational index | UNDP <http://hdr.undp.org/en/content/education-index> |
|  | *International* | Size | Total export value of G&T industry (billion USD) | ITC trade map [www.trademap.org](http://www.trademap.org) |
|  |  |  | Average export growth rate of G&T industry (%) |  |
| **Related and Supporting industries** | *Domestic* | Supporting industries | Cotton output (thousand tons) | USDA <http://apps.fas.usda.gov/psdonline/>  Vo and Wilder (2015)  Meador and Xinping (2014) |
|  |  |  | Yarn output (million tons) |  |
|  |  | Supporting infrastructures | Rail lines (total route – km) | World Bank <http://data.worldbank.org> |
|  |  |  | Roads, paved (% of total roads) | World Bank <http://data.worldbank.org> |
|  |  |  | ICT index | International Telecommunication Union [www.itu.int](http://www.itu.int) |
|  | *International* | Supporting industries | Cotton exports (thousand tons) | Vietnam Cotton and Spinning Association <http://www.vcosa.org.vn>  China Cotton Textile Association <http://www.ccta.org.cn/english/>  USDA <http://apps.fas.usda.gov/psdonline/> |
|  |  |  | Yarn and fabric exports (billion USD) |  |
|  |  | Supporting infrastructures | Container port traffics (TEU: 20 foot equivalent unit) | World Bank <http://data.worldbank.org>  World Bank <http://data.worldbank.org> |
|  |  |  | Air transport (registered carrier departures worldwide) |  |
| **Firm Strategy, Structure and Rivalry** | *Domestic* | Rivalry | Intensity of local competition | Schawab and Martín (2014) |
|  |  | Business context | World Bank DTF points | World Bank <http://www.doingbusiness.org/data/distance-to-frontier> |
|  | *International* | Rivalry | Market share of the country in G&T global market (%) | ITC Trade Map [www.trademap.org](http://www.trademap.org) |
|  |  | Business context | Average import tariff rate faced by G&T industry (%) | MAcMAp [www.macmap.org](http://www.macmap.org) |

*Source:* Developed by the authors based on Rugman and Verbeke (1993), Sardy and Fetscherin (2009), Balcarová (2010), Williams and Morgan (2010), Son and Kenji (2013)
