# Supplementary material for: A dynamic approach to assess international competitiveness of Vietnam’s garment and textile industry
Source: Springerplus. 2016 Feb 27;5:203. doi: 10.1186/s40064-016-1912-3 (PMC4769702; doi:10.1186/s40064-016-1912-3)
Supplement: Supplementary file 2 — 10.1186/s40064-016-1912-3 Competitiveness index of Factor Conditions. [file 40064_2016_1912_MOESM2_ESM.docx]

## Additional file 2 Competitiveness index of Factor Conditions

| **Attributes** | **Variables** | | **Proxies** | **Vietnam (%)** | **China (%)** |
| --- | --- | --- | --- | --- | --- |
| **Factor Conditions** | *Domestic* | Basic factors | Wage of worker in G&T industry (USD/h) | 358.11 | 100 |
|  |  |  | Number of workers and laborers in G&T industry (million people) | 10.87 | 100 |
|  |  |  | Labor productivity in G&T industry (shirts/worker/day) | 43.75 | 100 |
|  |  | Advanced factors | R&D expenditure (% of GDP) | 71.15 | 100 |
|  | *International* | Advanced factors | Manufacturing inward FDI flows (billion USD) | 3.76 | 100 |
|  |  |  | Manufacturing outward FDI flows (billion USD) | 2.72 | 100 |

Source: Authors' calculations
