# Supplementary material for: A dynamic approach to assess international competitiveness of Vietnam’s garment and textile industry
Source: Springerplus. 2016 Feb 27;5:203. doi: 10.1186/s40064-016-1912-3 (PMC4769702; doi:10.1186/s40064-016-1912-3)
Supplement: Supplementary file 3 — 10.1186/s40064-016-1912-3 Competitiveness index of Demand Conditions. [file 40064_2016_1912_MOESM3_ESM.docx]

**Additional File 3 Competitiveness index of Demand Conditions**

| **Attributes** | **Variables** | | **Proxies** | **Vietnam (%)** | **China (%)** |
| --- | --- | --- | --- | --- | --- |
| **Demand Conditions** | *Domestic* | Size | Total population (million people) | 6.65 | 100 |
|  |  |  | GDP (billion USD) | 1.80 | 100 |
|  |  |  | Employment rate (%) | 111.76 | 100 |
|  |  | Sophistication | GDP per capita (USD) | 27.03 | 100 |
|  |  |  | Household rate of expenditure on G&T out of gross income (%) | 45.83 | 100 |
|  |  |  | Educational index | 84.10 | 100 |
|  | *International* | Size | Total export value of G&T industry (billion USD) | 9.10 | 100 |
|  |  |  | Average export growth rate of G&T industry (%) | 260.81 | 100 |

Source: Authors' calculations
