# Supplementary material for: A dynamic approach to assess international competitiveness of Vietnam’s garment and textile industry
Source: Springerplus. 2016 Feb 27;5:203. doi: 10.1186/s40064-016-1912-3 (PMC4769702; doi:10.1186/s40064-016-1912-3)
Supplement: Supplementary file 4 — 10.1186/s40064-016-1912-3 Competitiveness index of Related and Supporting Industries. [file 40064_2016_1912_MOESM4_ESM.docx]

**Additional file 4 Competitiveness index of Related and Supporting Industries**

| **Attributes** | **Variables** | | **Proxies** | **Vietnam (%)** | **China (%)** |
| --- | --- | --- | --- | --- | --- |
| **Related and Supporting industries** | *Domestic* | Supporting industries | Cotton output (thousand tons) | 0.02 | 100 |
|  |  |  | Yarn output (million tons) | 2.25 | 100 |
|  |  | Supporting infrastructures | Rail lines (total route – km) | 3.54 | 100 |
|  |  |  | Roads, paved (% of total roads) | 78.16 | 100 |
|  |  |  | ICT index | 88.15 | 100 |
|  | *International* | Supporting industries | Cotton exports (thousand tons) | 0.00 | 100 |
|  |  |  | Yarn and fabric exports (billion USD) | 3.05 | 100 |
|  |  | Supporting infrastructures | Container port traffics (TEU: 20 foot equivalent unit) | 4.77 | 100 |
|  |  |  | Air transport (registered carrier departures worldwide) | 4.31 | 100 |

Source: Authors' calculations
