# Supplementary material for: A dynamic approach to assess international competitiveness of Vietnam’s garment and textile industry
Source: Springerplus. 2016 Feb 27;5:203. doi: 10.1186/s40064-016-1912-3 (PMC4769702; doi:10.1186/s40064-016-1912-3)
Supplement: Supplementary file 5 — 10.1186/s40064-016-1912-3 Competitiveness index of Firm, Strategy, Structure and Rivalry. [file 40064_2016_1912_MOESM5_ESM.docx]

**Additional file 5 Competitiveness index of Firm, Strategy, Structure and Rivalry**

| **Attributes** | **Variables** |  | **Proxies** | **Vietnam (%)** | **China (%)** |
| --- | --- | --- | --- | --- | --- |
| **Firm Strategy, Structure and Rivalry** | *Domestic* | Rivalry | Intensity of local competition | 94.44 | 100 |
|  |  | Business context | World Bank DTF points | 102.94 | 100 |
|  | *International* | Rivalry | Market share of the country in G&T global market (%) | 9.11 | 100 |
|  |  | Business context | Average import tariff rate faced by G&T industry (%) | 103.23 | 100 |

Source: Authors' calculations
