# Supplementary material for: A dynamic approach to assess international competitiveness of Vietnam’s garment and textile industry
Source: Springerplus. 2016 Feb 27;5:203. doi: 10.1186/s40064-016-1912-3 (PMC4769702; doi:10.1186/s40064-016-1912-3)
Supplement: Supplementary file 6 — 10.1186/s40064-016-1912-3 Competitiveness index of the GDDM. [file 40064_2016_1912_MOESM6_ESM.docx]

**Additional file 6 Competiveness index of the GDDM**

|  | **Vietnam (%)** | **China (%)** |
| --- | --- | --- |
| **Factor Conditions** |  |  |
| Domestic | 120.97 | 100.00 |
| International | 3.24 | 100.00 |
| Global | 62.11 | 100.00 |
| **Demand Conditions** |  |  |
| Domestic | 46.19 | 100.00 |
| International | 134.95 | 100.00 |
| Global | 90.57 | 100.00 |
| **Related and Supporting Industries** |  |  |
| Domestic | 34.42 | 100.00 |
| International | 3.03 | 100.00 |
| Global | 18.73 | 100.00 |
| **Firm Strategy, Structures and Rivalry** |  |  |
| Domestic | 98.69 | 100.00 |
| International | 56.17 | 100.00 |
| Global | 77.43 | 100.00 |

Source: Authors' calculations
